# Osteoradionecrosis of the Jaw—Comparison between Bone and Soft Tissue Injury and Their Influence on Surgical Outcomes—A Retrospective Cohort Study

**DOI:** 10.3390/diagnostics13030366

**Published:** 2023-01-18

**Authors:** Oliver Ristow, Jan Lukas Birgel, Thomas Rückschloß, Thomas Held, Kristin Lang, Maximilian Smielowski, Sven Zittel, Julius Moratin, Maximilian Pilz, Michael Engel, Jürgen Hoffmann, Karl Semmelmayer

**Affiliations:** 1 Department of Oral and Cranio-Maxillofacial Surgery, Heidelberg University Hospital, Im Neuenheimer Feld 400, D-69120 Heidelberg, Germany; 2 Department of Radiation Oncology, Heidelberg University Hospital, Im Neuenheimer Feld 400, D-69120 Heidelberg, Germany; 3 Institute of Medical Biometry, Heidelberg University Hospital, Im Neuenheimer Feld 130.3, D-69120 Heidelberg, Germany

**Keywords:** osteoradionecrosis, ORN, soft tissue damage, soft tissue injury, microvascular reconstruction

## Abstract

Surgical therapy of osteoradionecrosis of the jaw (ORN) is challenging and requires treatment of the affected hard and soft tissue. To understand how tissue injury after irradiation influences surgical outcomes, the objective of this study was to find out whether (a) bone-related, (b) soft tissue-related, and (c) treatment-related parameters influence the surgical success of patients with ORN. A total of 175 patients (324 lesions) were included in this retrospective, single-center study. All patients were diagnosed with ORN and underwent surgical therapy. The primary outcome was complete soft tissue recovery (mucosa/skin) and the absence of symptoms 3 months after surgery. At the time of follow-up, 58% of patients (189 of 324 lesions) had intact intraoral or extraoral soft tissue. The extent of bone destruction had no effect on treatment success, whereas soft tissue injury due to fibrosis (OR: 0.344; CI 0.142–0.834; *p* = 0.01818) and xerostomia (OR: 0.163; CI 0.064–0.419; *p* = 0.00016) increased the probability of treatment failure. Soft tissue reconstruction with a microvascular graft improved therapeutic success compared to local wound closure (OR: 2.998; CI 1.371–6.555; *p* = 0.006). Thus, for the treatment of ORN, it is extremely important to pay attention not only to the extent of bone destruction but especially to soft tissue defects. Because the extent of soft tissue injury is a predictor for therapeutic success, it should influence the choice of surgical treatment.

## 1. Introduction

Head and neck cancer is the seventh most common cancer type overall and is a major challenge for healthcare professionals [1]. Therapy remains demanding due to the heterogeneous nature of these tumors and the complicated anatomy of the head and neck region [2]. The therapy of choice primarily depends on tumor stage and tumor localization and comprises surgery, radiotherapy, and systemic therapy. Tumors in the oral cavity are predominantly treated surgically, while radiotherapy is commonly used as adjuvant therapy and for unresectable tumors [2].

Especially when tumors are proximal to the mandible and maxilla, however, radiotherapy carries a moderate risk of osteoradionecrosis (ORN) of the jaws, which can be a serious and devastating complication [2]. ORN manifests itself as exposed, irradiated bone that has lost its mucosal or skin lining, and occurs in the absence of a persistent or recurrent tumor [3]. The prevalence of ORN following radiotherapy is thought to vary between 5 and 15%, with wide variability reported in the literature. However, it is assumed to occur more often after radiation of the mandible compared to the maxilla [3]. Furthermore, it is often accompanied by recurring infections that include swelling, chronic dysphagia, and trismus and it can therefore strongly influence the quality of life of the affected patients [4]. Thus, it is of utmost importance to come to an early and targeted diagnosis, as quick surgical therapy is needed to maximize the restoration of mucosal integrity while keeping bone and tooth loss at a minimum.

The available data suggest a huge spread in the reported treatment success rates of ORN (ranging from 12% to 100%) [5,6,7,8,9]. However, across these reports, a large variety of different conservative or surgical treatment strategies with very different degrees of severity were used, while oftentimes even several consecutive surgical interventions were necessary, which makes these studies hard to compare.

Critically, most of these available studies focused on quantifying the extent of bone loss and investigated therapeutic strategies targeted at minimizing it [5,10,11]. However, radiotherapy also induces soft tissue injury, which is usually not considered in the preoperative planning and is also not always followed up on as a criterium for surgical success. This could be another explanation for the wide range of reported surgical success rates.

Interestingly, it has been shown that the extent of bone loss is not directly related to the extent of soft tissue injury and vice versa [12]. It is therefore not surprising that more and more groups are starting to consider both bone loss and soft tissue damage in their therapeutic algorithms for the treatment of ORN.

To understand the role of bone damage and soft tissue injury after irradiation and how they influence surgical outcomes, the objective of this study was to investigate how these factors impact the surgical treatment outcomes for ORN patients. The specific aims of this study were to assess whether (a) bone-related, (b) soft tissue-related, and (c) treatment-related parameters have a predictive influence on the success rate of surgical therapy of ORN.

## 2. Materials and Methods

### 2.1. Study Design

This study was designed as a retrospective cohort study and carried out at a single center (Department of Oral and Cranio-Maxillofacial Surgery, Heidelberg University Hospital, Heidelberg, Germany). The study was approved by the designated Research Ethics Board (S 329/2015) of the University of Heidelberg. We obtained the written consent of each patient in full accordance with ethical principles and the Declaration of Helsinki (version 2002). The manuscript was prepared according to the STROBE guidelines [13].

### 2.2. Study Sample

Study participants were recruited between 1 January 2010 and 31 December 2020 amongst patients who were diagnosed with or treated for ORN at our department. We diagnosed ORN according to the four following, commonly accepted criteria: (1) the occurrence of exposed irradiated bone that (2) lost its mucosal or skin lining, (3) failed to heal over a period of 3 months, and (4) occurred in the absence of any indication of persisting or recurrent tumor [3].

To be eligible for the study, patients had to fulfill the following inclusion criteria: (1) a history of malignancy (primary tumor or metastasis) in the head and neck region that had been treated with curative-intent or postoperative radiation therapy, (2) clinical and/or radiological signs of ORN, (3) a medical indication for surgical treatment of ORN, and (4) no preceding surgical attempts at treating the ORN lesion.

The exclusion criteria for patients were the following: (1) a patient’s refusal to receive surgical therapy for ORN, (2) any general medical precondition that did not allow for surgical therapy, (2) a history of antiresorptive treatment, (3) the presence of tumor disease in the affected region, (4) any missing postoperative follow-up examinations, (5) any missing preoperative radiologic data, and (6) patients were required to be at least 18 years of age.

### 2.3. Study Variables

The primary outcome was therapeutic success three months after surgical ORN treatment, which was defined as complete soft tissue (mucosal and skin) recovery and lack of any symptoms, such as swelling and pain.

The predictor variables were (1) the extent of bone destruction as defined radiologically by the following parameters: horizontal extension of osteolysis (larger or smaller than one alveolar compartment), vertical extension (more or less than the alveolar ridge), and the presence of hypersclerosis (yes/no); (2) the extent and type of soft tissue injury, defined as fibrosis, xerostomia, or mucositis (yes/no); and (3) the type of surgical therapy chosen, including details about the ablative (with/without continuity resection) or reconstructive approach (local wound closure, tissue reconstruction using microvascular free grafts with/without bone reconstruction).

Demographics and baseline characteristics, details about the underlying disease, and the parameters of radiotherapy were collected as secondary variables.

### 2.4. Surgical Protocol

All surgical treatments were performed under general anesthesia by the same team of experienced oral- and maxillofacial surgeons. All patients were pre-treated with oral antibiotics (Amoxicillin/clavulanic acid 875/125 mg 1-0-1; GlaxoSmithKline Consumer Healthcare GmbH & Co. KG, Munich, Germany) to achieve preoperative infection control for at least 10 days (and longer if necessary). In patients who reported a history of hypersensitivity to penicillin or a penicillin allergy, clindamycin 600 mg 1-1-1 (1A Pharma GmbH, Holzkirchen, Germany) was used instead. Additionally, all patients used an antimicrobial mouth wash (0.2% chlorhexidine solution; GlaxoSmithKline Consumer Healthcare GmbH & Co. KG, Munich, Germany) three times a day, starting with the first consultation, until removal of the sutures (10 to 14 days after surgery). All patients remained hospitalized for at least seven days after the surgery for administration of intravenous antibiotics and nutrition by gastric tube. In cases of extensive microvascular reconstruction, hospitalization was adapted to patient-specific needs. Clinical follow-up examinations were performed at three time points by investigators who had been trained in ORN treatment: between 10 and 14 days after surgery (coinciding with removal of sutures), 4 weeks, and 3 months postoperatively.

### 2.5. Statistical Protocol

Statistical analysis was performed with R version 4.1.1. For comparisons between the two groups (success yes/no) on patient level, chi-square tests were performed. To compare the two groups on case-level, mixed logistic regression models were fitted by using the respective patient as random effect. Differences were considered statistically significant when the *p*-value was lower than 5% (*p* < 0.05).

## 3. Results

### 3.1. Study Sample

A total of 175 patients matched our inclusion criteria and participated in the study. In this patient pool, we diagnosed a total of 324 ORN sites, of which 49 (15%) were located in the maxilla and 275 (85%) in the mandible. Baseline characteristics are summarized in Table 1. The overall mean patient age at the time of initial diagnosis of ORN was 70.1 years (Q1–Q3: 56–74 years). We had 52 female (30%) and 123 male (70%) patients in the cohort; no patient qualified as non-binary. We found that squamous cell carcinoma was by far the dominant tumor type (145/175; 83%), while adenoid-cystic carcinoma (20/175; 11%), mucoepidermoid carcinoma (20/175; 11%), Cancer of Unknown Primary (1/175; 1%), sarcoma (1/175; 1%), neuroblastoma (1/175; 1%), and metastasis (2/175; 1%) were observed less frequently. These tumors were located in the oropharynx (49/175; 28%), tongue (29/175; 17%), floor of mouth (28/175; 16%), mandible (21/175: 12%), maxilla (15/175; 9%), other (15/175; 9%), paranasal sinus 6 (3%), neck 5 (3%), parotid gland: 4 (2%), nasopharynx: 3/175 (2%). Tumor size was distributed as follows across our cohort: Tx: 3 (2%), T1: 22 (13%), T2: 63 (37%), T3: 31 (18%), T4: 56 (33%). Nodal status was distributed as follows: Nx: 3 (2%), N0: 66 (38%), N1: 35 (20%), N2: 64 (37%), N3: 7 (4%).

We found that none of these baseline characteristics correlated significantly with the primary outcome of the study (see below).

### 3.2. Primary Outcome

When we determined the primary outcome of the study at the three-month postoperative examination, we found that soft tissue was intact in 58% (189/324) of ORN lesions, which was therefore considered a positive primary outcome (see Methods). Conversely, 42% (135/324) of lesions still showed evidence of intraoral mucosal and/or extraoral skin defects and this was therefore considered a negative primary outcome.

First, we found that two parameters of the predictor variable “soft tissue injury”, were associated with a significant negative impact on primary outcome. While patients with fibrosis had a negative outcome in 51% (74/144) of lesions, patients without fibrosis had a negative outcome in only 44% (61/180) of lesions (OR: 0.344; CI 0.142–0.834; *p* = 0.01818).

Critically, we found an even clearer trend in patients with xerostomia, in which 64% (62/97) of lesions resulted in a negative outcome, which is a striking difference when compared with lesions in patients without xerostomia, of which only 32% (73/227) resulted in a negative outcome (OR: 0.163; CI 0.064–0.419; *p* = 0.00016).

By contrast, mucositis did not show a statistically significant influence on the therapeutic success, possibly due to a lower sample size. While 59% (19/32) of lesions in patients with mucositis led to a negative outcome, 40% (116/292) of lesions in patients without mucositis led to a negative outcome (OR: 0.518; CI 0.145–1.854; *p* = 0.31218).

Next, we investigated the influence of radiologically defined bone destruction on primary outcome. Importantly, we found that the radiological predictor variable “hypersclerosis” had a significant negative correlation with primary outcome (OR: 0.245; CI 0.075–0.795, *p* = 0.0192).

By contrast, we found that the predictor variable “extent of bone tissue destruction” had no significant influence on primary outcome by performing a chi-square test of independence to examine the relation between horizontal extension of the ORN (≤one alveolar compartment vs. >1 alveolar compartment) and primary outcome. The relation between these variables was not significant: *X*^2^ (1, *N* = 313) = 1.4121, *p* = 0.235. We obtained similar results when considering the vertical extension of the ORN: lesions larger than the alveolar ridge could not be associated with a worse treatment success compared to smaller lesions whose extent was less than the alveolar ridge (*X*^2^ (1, *N* = 258) = 0.1629, *p* = 0. 687).

Interestingly, there was also no significant correlation between the predictor variable “bone tissue destruction” and “soft tissue injury” (*p* > 0.05).

Finally, we investigated whether the predictor variable “surgical therapy” had any influence on the primary outcome. We found that neither local bone resections (closed: 55%, 127/230; open: 48%, 103/230) nor continuity resections (closed: 66%, 62/94; open: 34%. 32/94) (*p* = 0.075) resulted in significantly different surgical success rates. Importantly, however, soft tissue reconstruction with a microvascular graft (closed: 72%, 54/75; open: 28%, 21/75) improved the primary outcome when compared to local wound closure (closed: 54%, 135/249; open: 45%, 114/249) (OR: 2.998; CI 1.371–6.555; *p* = 0.006).

**Figure 1:**
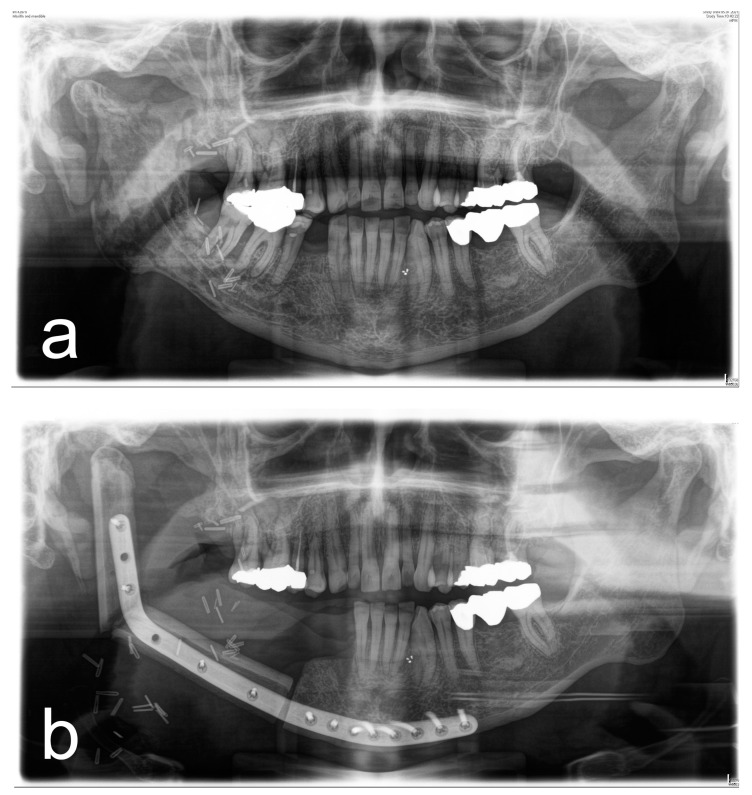
(**a**) Panoramic X-ray of a patient with extensive ORN of the mandible. (**b**) Postoperative panoramic X-ray of the same patient after ablative and reconstructive surgical therapy of ORN using a vascularized 2-segment fibula flap with intraoral soft-tissue transfer and patient-specific implant.

**Figure 2:**
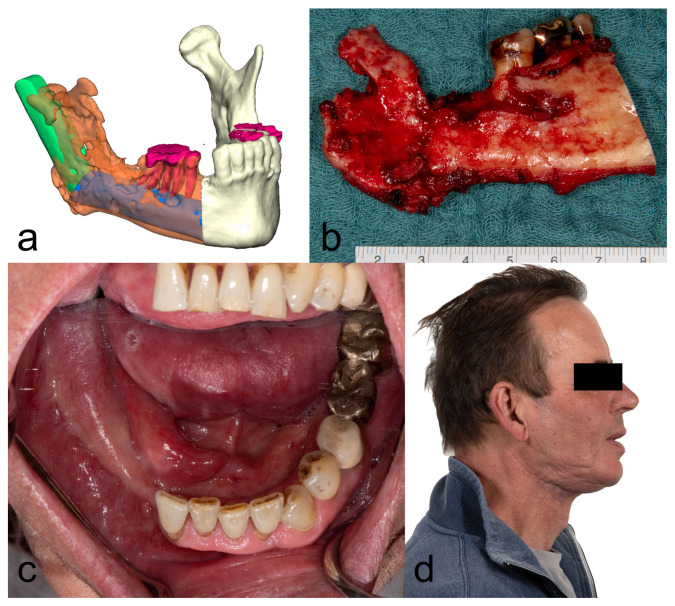
Clinical photos of the patient from Figure 1. (**a**) Digital treatment planning showing the aimed extent of the resection and position of the vascularized 2-segment fibula flap. (**b**) Surgical specimen on which the destruction of the bone can be clearly seen. (**c**) Intraorally placed skin paddle of the fibular graft for reconstruction of the extensive soft tissue defect (three months after surgery). (**d**) Lateral view three months after surgery.

**Figure 3:**
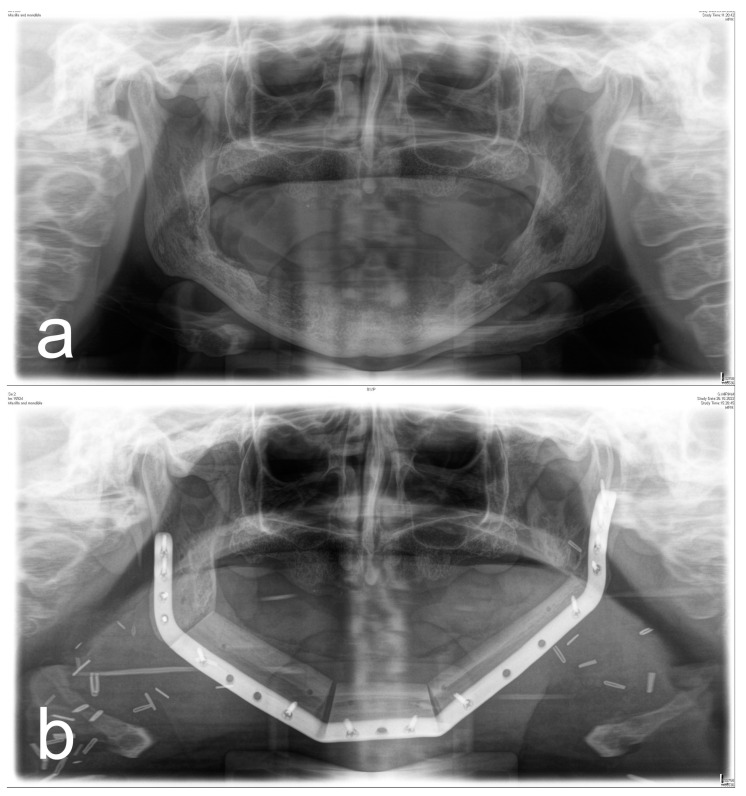
(**a**) Panoramic X-ray of a patient with extensive ORN of the mandible. (**b**) Postoperative panoramic X-ray of the same patient after ablative and reconstructive surgical therapy of ORN using a vascularized 3-segment fibula flap with an extraoral soft tissue transfer and a patient-specific implant.

**Figure 4:**
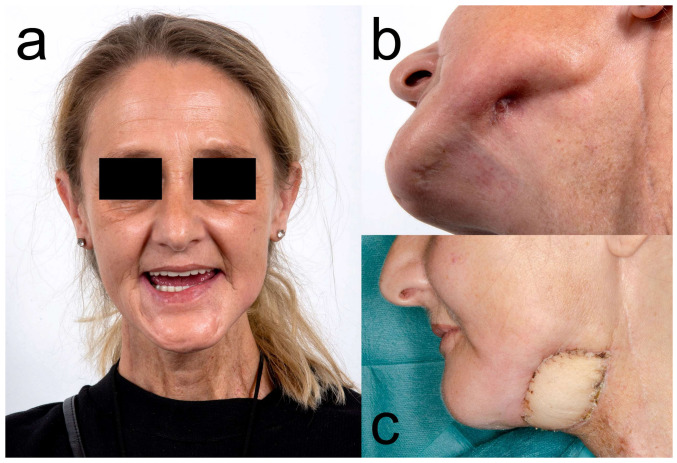
Clinical photos of the patient from Figure 3. (**a**) Preoperative situation with severely limited mouth opening. (**b**) Preoperative situation with extensive fibrotic soft tissue and extraoral cutaneous fistula on the left mandible. (**c**) Extraorally placed skin paddle of the fibular graft for reconstruction of the skin at the former site of cutaneous fistulae (one month after surgery).

Figure 1, Figure 2, Figure 3 and Figure 4 illustrate two cases of advanced mandibular resection with severe hard and soft tissue injury followed by reconstruction using a vascularized fibula flap. This graft allows reconstruction of bone and soft tissue, the latter of which can be used to replace extraoral skin (Figure 2) or intraoral mucosa (Figure 4).

### 3.3. Secondary Outcome

Considering the type of radiotherapy, 32 patients received 3D-CRT, whereas 86 patients received IMRT. Data on the type of radiotherapy were missing in 57 patients. We found that 3D-CRT treatment (*p* = 0.034) was correlated with therapeutic failure when compared to IMRT. Unsurprisingly, we also found that smoking had a negative effect on primary outcome (*p* = 0.007). By contrast, history of alcohol use (*p* = 0.339), body mass index (*p* = 0.397), diabetes mellitus (*p* = 0.477), tumor entity (*p* = 0.271), primary tumor site (*p* = 0.841), T-stage (*p* = 0.653), nodal status (*p* = 0.939), radiation dose (*p* = 0.267), concomitant immuno-/chemotherapy (*p* = 0.984), time between completion of RT and initial diagnosis of ORN (*p* = 0.237), ORN site (*p* = 0.554), and duration of perioperative antibiotic treatment (*p* = 0.508) had no significant effect on the outcome.

## 4. Discussion

The purpose of this study was to identify predictors of therapeutic success in the surgical management of ORN. The investigators hypothesized that regardless of the extent of bone destruction, it could be the soft tissue injury that has the greatest impact on surgical success. Therefore, the specific aims of the study were to assess whether (a) bone-related, (b) soft tissue-related, and (c) treatment-related parameters have a predictive influence on the success rate of surgical therapy of ORN.

The results of the study showed that the extent of soft tissue injury (especially fibrosis and xerostomia) is a predictor of therapeutic success and should therefore influence the choice of surgical treatment. Furthermore, we found that resections followed by free soft tissue transfer were clearly superior to local plastic wound closure.

It is of utmost importance to correctly diagnose the differential diagnosis of ORN at an early stage after radiotherapy and during regular tumor follow-up care. We assume that an early diagnosis and thus an early surgical intervention prevents the silent progression of the disease and has a positive influence on the extent of bone loss and thus on tooth loss. These factors influence dental rehabilitation and quality of life.

However, the correct choice of therapeutic strategy is just as important as an early diagnosis. For this purpose, a stage-dependent therapy is desirable. Nevertheless, most staging systems focus on the extent and degree of bone destruction [3,14,15]. However, there are no diagnostic methods that can accurately assess the extent of ORN. In our experience, the transition between post-radiogenic tissue adaptation, inflammatory overlay, and necrotic destruction is fluid, and making a correct estimation of its extent very difficult. Furthermore, tumor recurrence as a possible differential diagnosis is hard to distinguish from ORN.

In the present study, we demonstrated a significant correlation between surgical success and the extent of sclerosis. The literature suggests that the extent of bone-sclerosis is not equal to the extent of necrosis; instead, it usually extends beyond the truly dead areas. Rather, sclerosis seems to be a result of compacted centralization of the bone and depends on the mechanisms that serve to protect the bone. Thus, to resect all sclerosing portions would be an overtreatment.

In the present study, the horizontal and vertical dimension of bone destruction, in terms of osteolysis and sequestration, had no significant influence on the surgical outcome. The reason for this could be that a more radical surgical procedure is chosen for larger defects. Surgical options range from sequestrectomy and alveolectomy with primary wound closure to a large radical resection in patients with extensive bone and soft tissue defects followed by microvascular reconstruction [16]. According to the data available, the latter appears to be particularly successful [17].

By comparison, minor surgeries seem to have much poorer results and often require multiple procedures. While it is desirable to achieve restitution with a single surgical procedure, mandible continuity resection cannot be the correct choice for all patients. Interestingly, the data in the present study do not support the assumption that radical resection is always superior, as there was no significant difference on the therapeutic outcome between cases that are being locally resected and cases that demand a major bone resection with loss of continuity.

In summary, the aim must be to detect ORN as early as possible, correctly assess its extent, and remove as much bone as necessary but as little as possible. This requires knowledge of predictive factors [18,19]. Once these are identified, they can be incorporated into surgical planning.

One of these possible predictors is the handling of the soft tissue. The data of this study showed that whenever a free soft-tissue flap was used, it had a significantly positive impact on the therapeutic outcome. In this context, extensive resections often require free flap reconstruction. In addition to the extensive removal of the necrotic bone made possible by the planned reconstruction, healthy soft tissue can be introduced into the defect. In our view, this healthy tissue enables a tension-free wound healing and therefore leads to increased therapeutic success.

This goes in line with Ma et al., whose retrospective study of 47 patients with advanced ORN suggests that bone alteration alone appears to be a poor predictor of therapeutic success [12]. Their data show that soft tissue injury especially has a predictive value for surgical success. Therefore, the authors conclude that surgeons should be aware of the significance in assessing both soft tissue injury and bone destruction, include both factors in their therapeutic strategy, and thus deal with both during surgery. It is important to note that the extent of bone destruction in Ma et al.’s data did not correlate with the extent of soft tissue injury, which goes in line with the results of the present study. Additionally, our data showed that smoking has a negative effect on the therapeutic outcome, which further supports the importance of soft tissue healing in the treatment of ORN.

The importance of radio-induced fibrosis for surgical success may explain why the overall therapeutic outcome is much worse in ORN compared to medication-related osteonecrosis of the jaw (MRONJ), for which success rates of up to 90% have been reported in experienced centers [20]. However, soft tissue injury plays only a minor role in patients with MRONJ, as local soft tissue is sufficiently available for local multilayer closure techniques after stabilization of soft tissue toxicity and infection with anti-inflammatory and antibiotic drugs [21,22,23].

The role of adjunctive antibiotic therapy has also been critically discussed. This is mainly because the pathogenesis mechanism leading to ORN has not yet been definitively clarified. Whether an infection leads to the irradiated bone developing ORN or whether the ORN bone leads to a bacterial influx due to the destruction of the soft tissue remains to be determined. However, it is clear from much of the empirical data that the additional infection leads to ORN progression and therefore to a worsening of symptoms for the patient. By using conservative therapeutic regimens, i.e., antibiotics and local antimicrobial rinsing, an infected ORN can often be converted to a non-infected ORN. Thus, antibiotic treatment is useful and can stabilize the disease and lead to a reduction in symptoms. Conservative treatment strategies might be a treatment option if surgery is not possible due to the underlying disease of the patient. It cannot, however, be seen as an absolute alternative therapy to surgery. Unnecessary delay should be avoided as there is a risk of silent (asymptomatic) progression of the disease. The effect of antibiotic therapy on necrotic bone is largely unclear. Only a few studies have looked at the actual concentration of antibiotics in necrotic bone [24,25]. These studies describe low but sufficient concentrations of beta-lactam antibiotics to eradicate most bacteria, such as Streptococci and Staphylococci, which are typically present in ORN. In our opinion, antibiotic therapy not only leads to reduction in symptoms for the patient but overall supports the surgical procedure by providing a low-inflammatory situation, especially in the soft tissue. It therefore assists wound healing and thus the success of the therapy.

As the data of this study showed, local wound closure is not reliably successful in ORN. This is especially true if the degree of fibrosis is high. Irrespective of the level of bone destruction, the soft tissue must be considered in therapy planning. If necessary, free soft tissue grafts should be used generously in addition to surgical removal of fibrosis [9]. This may be part of a bone reconstruction effort after continuity interruption or may be required independently and/or in conjunction. In addition, vascularity (dermal/subdermal circulation) and elasticity at the junction between the soft tissue graft and the fibrosed local tissue (mucosa or skin) should be checked during surgery. It is not uncommon for postoperative wound dehiscence to occur at the junctions between local soft tissue and tissue of a free flap, affecting the overall surgical outcome. A solution to this may be (i) subcutaneous scar release to loosen the indurations and improve subdermal blood flow, (ii) ensuring sufficient thickness of the soft tissue graft to tolerate compromised superficial wound healing, or in the last consequence (iii) resection of the fibrotic tissue and reconstruction by a sufficiently sized soft tissue transfer.

The present study considered a heterogeneous collective with different severities of ORN. This could be interpreted as a limitation of the study. However, the few studies that have examined the significance of soft tissue injury mainly examined severe ORN, leading to some selection bias. Since the available staging systems are based on bone destruction and a significant correlation between bone destruction and soft tissue injury is not apparent, it is important to consider all stages, as the soft tissue situation is crucial for the therapeutic success. In future prospective studies, stratification by stages would be of interest, possibly considering soft tissue status.

The retrospective design is a limitation of the study. However, this retrospective study stands out due to its large number of cases treated by a limited team in a single special consultation center for osteonecrosis. This standardizes treatment procedures and documentation and can be seen as a quality feature.

## 5. Conclusions

Successful therapy of ORN is a difficult challenge. We emphasized the importance of an early diagnosis and early surgical therapy to prevent silent progression of the disease and to minimize loss of bone, teeth, and quality of life. In surgical planning, the focus should not be limited to the bone defect but should also consider the soft tissue injury. Regardless of how extensive the bone damage is, a free soft tissue transfer should be considered.

## Figures and Tables

**Table 1:** Baseline characteristics of the assessed patients with osteoradionecrosis.

Variable	Closed Mucosa/Skin N = 96	Open Mucosa/Skin N = 79	Total N = 175
**Gender**			
Female	32	20	52
Male	64	59	123
**Disease**			
SCC	80	65	145
ACC	8	12	20
MEC	4	1	5
CUP	1	0	1
Sarcoma	0	1	1
Neuroblastoma	1	0	1
Metastasis	2	0	2
**Smoking**			
Yes	45	53	98
No	51	26	77
**Alcohol**			
Yes	31	31	62
No	65	48	113
**Dose of Radiation**			
Mean	68 Gy	69 Gy	69 Gy
Standard Deviation	11 Gy	10 Gy	11 Gy
**Time from the end of Radiotherapy until ORN onset**			
Median	870 days	676 days	714 days
Q1–Q3	306–2097 days	270–2026 days	299–2082 days

## Data Availability

The data presented in this study are available on request from the corresponding author.
